# Inherent promoter bidirectionality facilitates maintenance of sequence integrity and transcription of parasitic DNA in mammalian genomes

**DOI:** 10.1186/1471-2164-10-498

**Published:** 2009-10-27

**Authors:** Paul Kalitsis, Richard Saffery

**Affiliations:** 1 Chromosome and Chromatin Research, Murdoch Childrens Research Institute, Royal Children's Hospital, Parkville, Melbourne 3052, Victoria, Australia; 2 Developmental Epigenetics, Murdoch Childrens Research Institute, Royal Children's Hospital, Parkville, Melbourne 3052, Victoria, Australia; 3 Department of Paediatrics, University of Melbourne, Parkville, Melbourne 3052, Victoria, Australia

## Abstract

**Background:**

Many mammalian genes are arranged in a bidirectional manner, sharing a common promoter and regulatory elements. This is especially true for promoters containing a CpG island, usually unmethylated and associated with an 'open' or accessible chromatin structure. In evolutionary terms, a primary function of genomic methylation is postulated to entail protection of the host genome from the disruption associated with activity of parasitic or transposable elements. These are usually epigenetically silenced following insertion into mammalian genomes, becoming sequence degenerate over time. Despite this, it is clear that many transposable element-derived DNAs have evaded host-mediated epigenetic silencing to remain expressed (domesticated) in mammalian genomes, several of which have demonstrated essential roles during mammalian development.

**Results:**

The current study provides evidence that many CpG island-associated promoters associated with single genes exhibit inherent bidirectionality, facilitating "hijack" by transposable elements to create novel antisense 'head-to-head' bidirectional gene pairs in the genome that facilitates escape from host-mediated epigenetic silencing. This is often associated with an increase in CpG island length and transcriptional activity in the antisense direction. From a list of over 60 predicted protein-coding genes derived from transposable elements in the human genome and 40 in the mouse, we have found that a significant proportion are orientated in a bidirectional manner with CpG associated regulatory regions.

**Conclusion:**

These data strongly suggest that the selective force that shields endogenous CpG-containing promoter from epigenetic silencing can extend to exogenous foreign DNA elements inserted in close proximity in the antisense orientation, with resulting transcription and maintenance of sequence integrity of such elements in the host genome. Over time, this may result in "domestication" of such elements to provide novel cellular and developmental functions.

## Background

The emergence of novel gene functions is an essential driving force behind the evolution of species. Many molecular mechanisms have been described that contribute to this process including gene duplication, exon shuffling, retroposition, transposable element insertion, lateral (horizontal) gene transfer, and gene fusion/fission events [1].

One of these mechanisms, transposable or mobile elements, are segments of DNA encoding genes that assist in DNA excision, replication and integration of the elements into new regions of the genome. Until recently transposable elements (TEs) have been considered parasitic or selfish DNAs that contribute little to the host organism [2,3]. These elements generally exist as neutrally evolving inactive DNA remnants that are epigenetically silenced by the host genome to prevent transcription and subsequent transposition [4,5]. Such elements are therefore subject to little selective pressure and subsequently acquire sequence variation (mutations) over time. However, it has recently been shown that some TEs escape host cell silencing to become 'domesticated' by host genomes resulting in the formation of novel genes [6-8]. Such domesticated elements are involved in many cellular and developmental functions including placental development, viral resistance, chromatin structure, DNA recombination and repair, gene regulation, apoptosis and brain development [8]. Given these facts, it appears that a complex evolutionary interplay exists between genomic silencing of transposon elements to prevent their proliferation, and co-option of transposon-encoded proteins to provide novel cellular functions in higher eukaryotic genomes.

In an attempt to understand and identify the molecular mechanism/s of domestication we have examined the number of such genes in the human and mouse genomes and analysed the features of their genomic insertions sites. We classified each according to gene/promoter structure, degree of conservation, expression profile, and transposable element type.

Whereas previous studies have suggested that the majority of transposable element domestication arises via a gene fusion event or genomic insertion in close proximity to a cryptic promoter [1,8,9], our analysis has revealed that only a small proportion of co-opted TE-derived genes arise from such events. Rather, we have identified a significant number of domesticated TEs that share a promoter with a neighbouring gene in a head-to-head, or bidirectional arrangement. We have further examined the role of CpG rich 'inherently bidirectional' promoters in the insertion of foreign elements in recently evolved genes and experimental systems.

## Results

### Domestication of protein-coding transposable elements in mammalian genomes

Recent studies have surveyed eukaryotic genomes for the presence of TEs that have been co-opted by their host genome [7-10]. In the human genome it has been estimated that approximately 0.1% of all protein-coding genes contain (at least) part of a TE, however little data are available related to the potential mechanisms leading to TE co-option [9]. We have utilised updated genome annotation and genome-wide expression data to expand this analysis to further examine the co-option status of inserted transposable elements by mammalian genomes (Tables 1 and 2 and Additional file 1 and 2). Since a high proportion of protein-coding genes in both humans and mouse contain small insertions from common TEs such as Alu and B1 type elements, we limited our survey to genes with protein coding regions of at least 30% sequence length homology to a known TE (see Materials Methods).

**Table 1:** Survey of human domesticated genes^a^

**Repeat family**	**Repeat type**	**Number**	**Chimeric**	**Bidirectional**	**Bidirectional with CpG island**
DNA transposons	Tigger	13	2	8	8
	PiggyBac	5	2	1	1
	THAP (P-element)	12	2	6	6
	HSMAR (mariner)	1	1	0	0
	Others	11	2	3	3
Retrotransposons	Paraneoplastic antigens	8	0	2	2
	Ty3/gypsy	8	0	2	2
	Others	3	1	1	1
Endogenous retroviruses	Syncytin	2	0	0	0

Total		63	10 (15.9%)	23 (36.5%)	23

^a ^For a more detailed survey of domesticated TEs in the human genome see Additional file 1

**Table 2:** Survey of mouse domesticated genes^a^

**Repeat family**	**Repeat type**	**Number**	**Chimeric**	**Bidirectional**	**Bidirectinal with CpG island**
DNA transposons	Tigger	8	2	2	2
	PiggyBac	2	1	0	0
	THAP (P-element)	7	2	1	1
	Others	6	2	3	3
Retrotransposons	Paraneoplastic antigens	7	0	1	1
	Ty3/gypsy	8	0	2	2
	Others	3	0	1	1
Endogenous retroviruses	Syncytin	2	0	0	0
	Others	2	0	2	2

Total		45	7 (15.6%)	12 (26.7%)	12

^a ^For a more detailed survey of domesticated TEs in the mouse genome see Additional file 2

Using these selection criteria, we identified a total of 63 transcribed protein-coding genes within the human genome derived from previously characterised TEs. These can be grouped into three main classes: DNA transposons, retrotransposons and endogenous retroviruses. The most abundant group are DNA transposons (n = 42) followed by retrotransposons (n = 19) and endogenous retroviruses (n = 2) (Table 1 and Additional file 1). Of note, all of these elements contain intact open reading frames and evidence of transcriptional activity, suggesting production of functional proteins. (Additional files 1 and 2). Fewer TE-derived genes were identified in the mouse genome (45 compared with 63). However, a similar distribution of TE classes was observed for mouse with 23 DNA transposons, 18 retrotransposons and 4 endogenous retroviruses.

### A link between TE domestication and bidirectional promoters?

**Figure 1:**
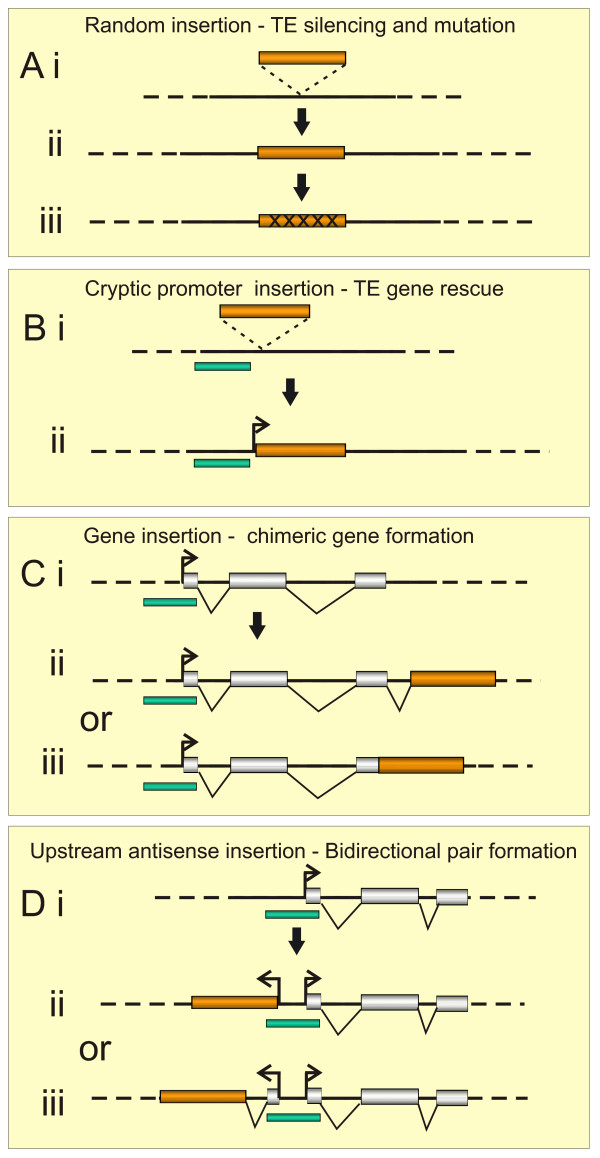
**Molecular models for transposable element co-option and domestication**. (A) Transposable elements often insert into intergenic or intronic regions but are generally transcriptionally silenced through epigenetic modification. Over time mutations accumulate due to a lack of selective pressure to maintain sequences intact. If the TE contains sequences that drive transcription it may remain active if insertion takes place in a genomic location that favours expression over TE repression and silencing. (B) TE may insert into a region of the genome with inherent transcriptional activity or in close proximity to a cryptic CpG rich promoter (green box) that may result in TE avoiding epigenetic silencing to remain active. (C) Less frequently, TEs may insert downstream of the transcription start site of a host gene (i) either into an intron, or (ii) exon (iii) with the potential to create novel chimeric genes and/or proteins. (D) Insertion of a TE upstream of a host gene in an antisense direction. (i) Pre-insertion genomic environment displaying a typical multi-exon unidirectional gene. (ii) Incoming TE may insert within 1 kb of a CpG island gene promoter with transcription initiating within the TE itself. (iii) Alternatively the TE may insert further away from host promoter but will form a novel gene with a new upstream exon and an intervening intron that is spliced during mRNA formation.

TE insertion into downstream regions (exons or introns) of annotated genes to create a chimeric gene has previously been reported (Figure 1 and Additional file 1 and 2) [9,11]. Of the 63 potential domesticated TE genes we have identified in the human genome, only 10 (15.9%) are likely to have arisen in this manner. Thus, 84% of the domesticated genes show an alternative mechanism of insertion. Further analysis of the genomic landscape associated with this subclass revealed a disproportionate number that appear to share a common promoter arranged in an antisense orientation (ie. bidirectional pair).

To test the frequency of this arrangement we compared the incidence of TE-associated bidirectionality with the human genome average of bidirectional gene pairs. Exact estimates of the number of bidirectional genes in the human and mouse genomes vary, between 9.8% (4,226 of 42,887 transcription units; [12]) and 11% of human genes (2,700 of 24,000 genes; [13]), and 8.9% of mouse transcriptional units (3,276 of 36,606) exist as part of a bidirectional pair, with transcription start sites less than 1 kb apart [12,13]. Using similar criteria we observed that 23 of the 63 (36.5%) co-opted TE genes exist as part of a bidirectional pair in humans which is significantly greater than the genome average of such an arrangement; χ^2 ^= 39.3, p < 0.0001 (Table 1 and Additional file 1). The mouse genome also contains a significantly higher proportion of co-opted genes in bidirectional pairs (12 of 45, 26.7%) when compared with the genome average (χ^2 ^= 17.5, p < 0.0001) (Table 2 and Additional file 2). In order to test whether the 30% TE similarity cut off used in our analysis preferentially identified genes with a reduced cDNA length relative to the genomic average, we calculated the mean cDNA length of bidirectional gene pairs relative to the genome-wide average. No significant difference between the two data sets was observed with a genome-wide mean cDNA length of 2465 bp (2130 bp median) [13] and a mean cDNA length of bidirectional pairs of 2352 bp (2086 bp median).

Between 77% [13] and 94% [12] of bidirectional promoters in the human genome contain a classical CpG island, defined as a stretch of DNA over 200 bp, with a G + C content of over 50%, and an observed/expected ratio of CG dinucleotides of over 0.6 [14]. This appears to be a specific feature of mammalian genomes [15]. The corresponding proportion for unidirectional promoters in the human genome is between 38% [13] and 60% [12] depending on whether annotated genes or transcriptional units are used as a reference. CpG island length of bidirectional promoters appears significantly longer than unidirectional promoters [12]. However CpG island size is not strictly a determinant of bidirectionality status [16]. In human and mouse genomes we found that all cases of bidirectional promoters associated with a domesticated TE contain a CpG island that shows some overlap of the first exons of both the host and domesticated TE genes (Table 1 and 2, and Additional files 1 and 2).

### Inherent bidirectionality and open chromatin of CpG islands - a chink in the defensive armour

Most CpG islands within the human genome exist in an unmethylated state and are associated with constitutively active genes [17,18]. Such a state is invariably associated with an open chromatin structure that is anticipated to render the underlying DNA accessible to chromatin-associated proteins involved in the transcriptional regulation. We postulated that the majority of promoters containing CpG islands within mammalian genomes show inherent bidirectional capacity which, coupled with their open chromatin state, renders them ideal target sites for the integration and subsequent transcription of TEs arranged in an antisense direction to the endogenous partner gene. We anticipated that the constitutive transcriptional state of the promoter region of the founding member of the pair has the additional effect of protecting the incoming parasitic DNA element from the endogenous gene silencing machinery that would normally render such insertions inactive and prevent their expression following integration.

To test whether CpG islands associated with unidirectional transcription units have inherent properties permissive for bidirectional transcription, we investigated the nature of trapped loci that have been characterised as part of the International Gene Trap Consortium (IGTC) in mouse embryonic stem cell lines [19]. We screened the database for the presence of anti-sense transcripts which overlapped or were upstream of exon 1 of an annotated gene. These were aligned for position and direction within the mouse genome. We selected anti-sense gene-trap tags according to the following selection criteria; overlapping with exon 1 or the predicted promoter, and within 2 kb upstream of the annotated transcription start site or CpG island. Sequence tags located downstream of the annotated transcription start sites and known bidirectional genes were excluded from analysis.

From a total of 7592 Ensembl genes trapped by the IGTC as of 8-May-2006 we identified 11 (0.145%) annotated genes or transcriptional units (TUs) associated with anti-sense trap-tags (Table 3 and Additional file 3). Of these, seven contain more than one tag in the anti-sense direction, and two loci are tagged with more than one type of gene-trap vector. Similar to the bi-directionally co-opted human and mouse TEs, all of the genes associated with anti-sense trap tags have an associated CpG island. Further evidence in support of antibiotic resistance gene cassette rescue by anti-sense transcription is the splicing of the resulting anti-sense transcript tags or rare ESTs, as observed for the *Daxx*, *Kif2a *and *Uba52 *genes. Cap analysis gene expression (CAGE) tags indicative of potential anti-sense transcription start sites [20] were also found at most sites of bidirectional gene-trap insertions (Table 3).

**Table 3:** Anti-sense rescue of gene trap cassettes in mouse ES cells^a^

**Gene**	**Anti-sense gene trap tags**	**CpG island**	**CAGE tag sense^a^**	**CAGE tag anti-sense^b^**
2610209A20Rik	1	yes	44	1
Atp5b	1	yes	3843	7
Daxx	2	yes	427	19
Fnbp4	12	yes	1170	0
Kif2a	2	yes	7	7
Kpnb1	4	yes	335	81
BC096391	2	yes	166	0
Ppm1b	3	yes	367	1
Rab30	1	yes	36	24
Top3b	1	yes	511	0
Uba52	7	yes	1743	4

^a ^See Additional file 3 for more details.

^b ^Cap analysis of gene expression (CAGE) tags were scored within 1 kb of the consensus start site of transcription. Sense and anti-sense CAGE tags are orientated with respect to the listed trapped gene.

Further data supporting an inherent bidirectionality of 'unidirectional' gene promoters in the human genome has also been described [13]. Of 56 random nonbidirectional promoters assayed for transcriptional activity *in vitro*, 52% were active in both directions. In contrast, 90% of 258 assayed bidirectional promoters were active in both directions. Data from this study also suggests that bidirectional promoters share a common DNA region necessary for transcription in both directions and therefore exist as inseparable functional units [13].

In combination, these data demonstrate the inherent capacity for bidirectional transcription of CpG island-associated promoters in mammals, adding support to our hypothesis that such sites are suitable for the opportunistic insertion and expression of foreign TE transcriptional units into mammalian genomes.

### TIGD1, a recently domesticated DNA-based transposable element

**Figure 2:**
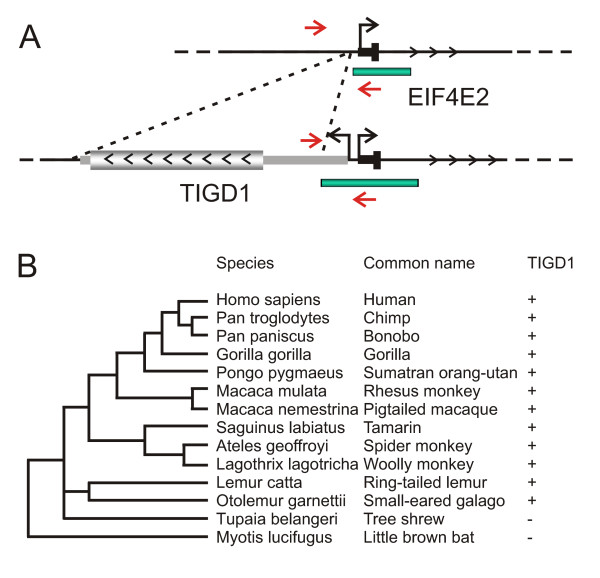
**Formation of the TIGD1 gene in primates**. (A) Schematic representation pre- and post- insertion of the TIGD1 ancestral TE with locations of amplification primers (red arrows) used to screen for the presence of the TIGD1 gene in mammalian species and CpG island (green bars). Non-coding exons are denoted by narrow boxes and coding exons by larger shaded boxes with the direction of transcription indicated by arrowheads. (B) Molecular phylogenetic analysis of the domestication of TIGD1 in primates and a summary of TIGD1 bioinformatic or PCR screening assay. (+) denotes the presence of the TIGD1 gene and (-) denotes the absence of this gene within specific species. Phylogenetic tree of primate lineage displayed.

*TIGD1 *is an example of a recent co-opted transposable element present in human and chimp genomes as part of a bidirectional gene pair. DNA sequence analysis reveals that TIGD1 retains the conserved catalytic DDE/D core but lacks one of the terminal inverted repeats necessary for transposition (data not shown) [21]. To further delineate the extent of conservation and estimate the time of acquisition of this element in evolution, we searched available genomic sequence datasets or performed PCR analysis on the intergenic junction region between the *TIGD1 *and *EIF4E2 *genes in a panel of primate and other mammalian genomic DNAs. *TIGD1 *was detected in all primate species including human, *Pan troglodytes *(chimp), *Pan paniscus *(bonobo chimp), *Gorilla gorilla*, *Pongo pygmaeus *(orangutan), *Macaca mulata *(rhesus monkey), *Macaca nemestrina *(pigtailed macaque), *Saguinus labiatus *(tamarin), *Ateles geoffroyi *(spider monkey), Otolemur garnetti (small-eared galago) and *Lemur catta *(ring-tailed lemur) (Figure 2 and Additional file 4). However, *TIGD1 *is absent in other mammalian groups examined including the tree shrew, *Tupaia belangeri *(Scandentia) and the little brown bat, *Myotis lucifugus *(Chiroptera), the next closet representative orders to primates. Therefore *TIGD1 *domestication occurred concurrently with the divergence of the primate lineage between 67 to 98 million years ago [22].

### Murine bidirectional endogenous retroviruses

**Figure 3:**
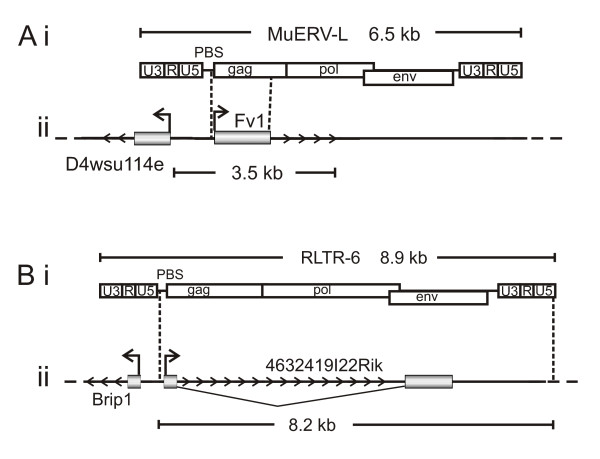
**Bidirectional insertion of mouse endogenous retroviruses to form 4632419I22Rik and Fv1 genes**. (Ai and Bi) Presumed parental endogenous retroviruses, MuERV-L and RLTR-6 with LTR domains (U3-R-U5), primer binding site (PBS) and open reading frames, gag, pol and env. (Aii and Bii) Structure of the domesticated 4632419I22Rik and Fv1 genes showing regions of homology to presumed ancestral ERV (dashed lines). Exons are denoted by grey boxes and direction of transcription is indicated with arrow heads.

The Friend virus susceptibility gene (Fv1) confers resistance against the murine leukaemia virus in mice [23,24]. This endogenous retrovirus appears to have been a recent co-option event, since it is not present in the rat genome. It shares homology with the gag domain of other endogenous mammalian retroviruses, especially the mouse MuERV-L (Figure 3). *Fv1 *exists in a bidirectional pairing with the *D4Wsu114e *gene (transcription start sites <250 bp apart). Although endogenous retroviruses are generally flanked by 5' and 3' LTR sequences (5'LTR sequence conferring promoter activity), no such promoter LTR elements appear to have contributed to the insertion and retention of this endogenous retroviral element.

**Figure 4:**
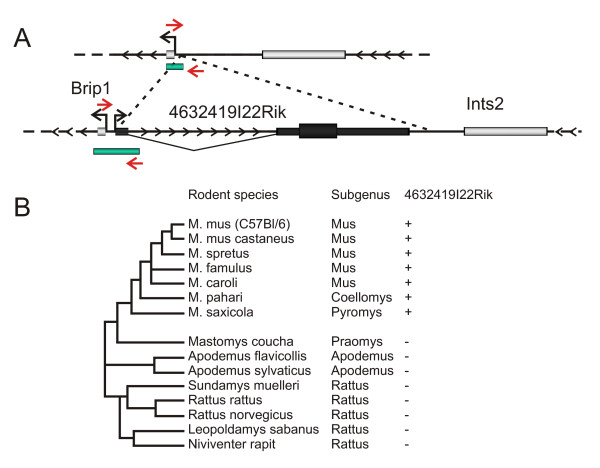
**Formation of the 4632419I22Rik gene in rodents**. (A) Schematic representation pre and post insertion of a 4632419I22Rik ancestral TE with locations of amplification primers (red arrows) used to screen for the presence or absence of the 4632419I22Rik gene in mammalian species and CpG island (green bars). Non-coding exons of 4632419I22Rik and neighbouring genes Brip1 and Ints2 are denoted by narrow boxes and coding exons by larger shaded boxes with the direction of transcription indicated by arrowheads. (B) Molecular phylogenetic analysis of the domestication of 4632419I22Rik in rodents and a summary of 4632419I22Rik screening assay. (+) denotes the presence of the 4632419I22Rik gene and (-) denotes the absence of this gene within specific species. Phylogenetic tree of rodent lineage is displayed.

Another mouse-specific endogenous retrovirus of unknown function that has recently been co-opted is the *4632419I22Rik *gene. This exists as part of a bidirectional pair with *Brip1*, (transcription start sites 170 bp apart). PCR amplification of a panel of rodent species revealed the presence of this gene in all *Mus *species including; *M. musculus*, *M. musculus *castaneus, *M. spretus*, *M. famulus*, *M. caroli, M. pahari, and M. saxicola *(Figure 4 and Additional file 5). These results suggest that *4632419I22Rik *appeared with the *Mus *lineage, after the divergence of the *Rattus sensu lato *and *Praomyini *(containing Mastomys) clades [25]. Similar to the *Fv1 *insertion, *4632419I22Rik *does not include a 5'LTR sequence (Figure 3).

### Retroposed genes

Retroposed genes usually arise from the reverse transcription of a host cell RNA with subsequent integration of the intronless cDNA into the host genome at a new location, usually in the absence of any upstream regulatory elements. This process is thought to be mediated by the by the reverse transcriptase enzyme of the L1 family of retrotransposons [1]. To investigate the degree to which transcribed retroposed genes are inserted and maintained through inherent bidirectional activity of CpG island promoters, we examined the genomic location of the top 20 transcribed retroposed genes in the human genome [26] (Additional file 6). Of these, six (30%) were found to be bidirectional (transcription start site within 1 kb of an annotated gene) and associated with a CpG island. When we extended our analysis to include the top 50 transcribed retrogenes, no additional instances of bidirectionality were observed suggesting a possible link between bidirectionality and transcriptional activity.

### Genomic environment - pre and post insertion

**Figure 5:**
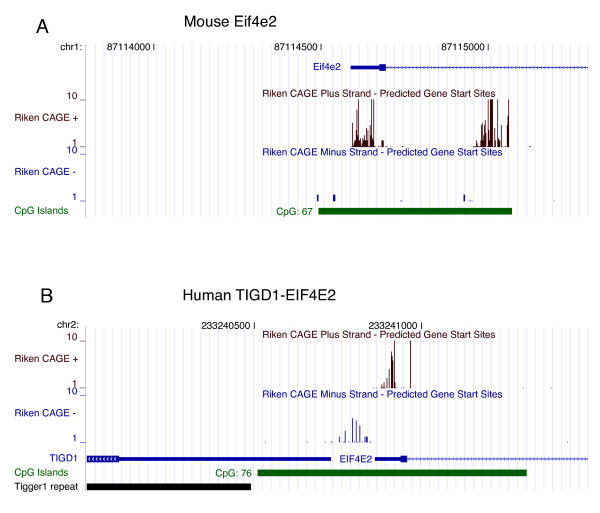
**Transcriptional activity and CpG island genomic environment, pre and post insertion of TIGD1**. (A) Promoter region of the mouse Eif4e2 gene shows a predominantly unidirectional grouping of cap analysis gene expression (CAGE) tag transcription start sites in the plus direction (brown vertical bars) together with some detectable activity in the minus direction (blue vertical bars). (B) After the insertion of the TIGD1 element in the primate lineage, the human TIGD1-EIF4E2 promoter region displays a lengthening of the CpG island and bidirectional transcription of the TIGD1 and EIF4E2 genes, blue and brown vertical bars, respectively. The Riken CAGE transcription start sites are overlayed on a 1,500 bp window for mouse and human genome assemblies mm5 and hg17, respectively.

To examine the genomic landscape before and after gene insertion we chose a group of recently domesticated genes that exist as part of novel bidirectional pairings with a common CpG island promoter. This list comprised two primate-specific genes (*TIGD1 *and *PGBD4*) and two mouse-specific genes (*Fv1 *and *4632419I22Rik*). Each ancestral gene promoter was anticipated to be unidirectional. However, two of the four genes display some degree of bidirectional activity as evidenced by CAGE and/or EST tags in species lacking the TE insertion element (Table 4 and Figure 5). TE insertion and maintenance of coding capacity appears to be associated with an increase in CpG island length for the *TIGD1-EIF4E2*, *4632419I22Rik-Brip1 *and *Fv1-D4Wsu114e *promoter regions, whereas the CpG island associated with *PGBD4-2900064A13Rik/C15ORF24 *remained unchanged following TE insertion (Table 4). Increasing size is associated with an increase in the number of CpG dinucleotides in the region closest to the newly co-opted gene. Interestingly, this is also associated with an increase in the ratio of antisense: sense (co-opted: host) CAGE tags associated with each CpG island region post TE insertion, suggesting an increasing capacity for antisense transcription or increasing transcript stability following TE insertion (Table 4 and Figure 5).

**Table 4:** Transcriptional activity, pre and post transposable element insertion

**Species**	**Host Gene**	**CpG Island Length (bp)**	**CAGE tags**	**Antisense** **Co-opted gene**	**CAGE tags**	**CAGE tag ratio**
mouse	Eif4e2	566	174, 205^a^	-	6, 5^a^	0.034, 0.024^a^
human	EIF4E2	806	173	TIGD1	51	0.29
						
mouse	2900064A13Rik	469	1736	-	0	0
human	C15ORF24	450	1509	PGBD4	7	0.0046
						
human	IIP45	652	18	-	1	0.056
rat	D4Wsu114e	250	9^b^	-	0^b^	0
mouse	D4Wsu114e	301	63	Fv1	19	0.3
						
human	BRIP1	242	9	-	0	0
rat	Brip1	211	6^b^	-	0^b^	0
mouse	Brip1	856	129	4632419I22Rik	312	2.4

^a ^Two transcriptional start sites that are approximately 400 bp apart

^b ^No CAGE data available for rat therefore ESTs were used instead

### Co-expression of human TE-bidirectional gene pairs

To examine whether the human TE-derived bidirectional gene pairs exhibit coordinate expression we performed a correlation analysis using publicly available GNF Gene Expression Atlas 2 data on 79 human tissues [27]. A Pearson correlation coefficient was calculated for nine gene pairs where both were included on the array and at least one of which exhibited detectable expression levels following array analysis (Additional file 7). Of the pairs examined 56% (5/9) showed a correlation coefficient of greater than 0.7 with 33% (3/9) showing a correlation value of over 0.8. This supports previous data showing bidirectional gene pairs are more likely to be co-expressed when compared with random gene pairs [13].

## Discussion

Most of the DNA that makes up complex genomes is parasitic in nature, derived from transposable elements acquired throughout evolution. Generally, these elements exist as neutrally evolving inactive DNA remnants (or fossils) silenced by the host genome over time. Relatively little attention has been paid to a minority of conserved TEs that retain protein coding capacity, and are expressed with varying degrees of cell-type specificities. Many of these are conserved across phylogeny, suggesting a degree of selective pressure over time that may be indicative of functional importance [21,28].

The process of "domestication" of such transposable element-encoded proteins is just beginning to be appreciated and begins with a co-option (integration and expression) of a TE into the host genome. Numerous examples of such elements contributing to the acquisition of specific novel host cell functions have been described in a variety of organisms. Some of these examples include regulation of light signalling in Arabidopsis [29], mammalian neuronal development [30] and resistance to viruses in mice [23]. A recent study highlighting this phenomenon demonstrated that inactivation of a retrotransposon-like element in mice leads to embryonic lethality due to placental defects [31]. Thus, a complex evolutionary interplay appears to exist between genomic silencing of transposon elements to prevent their proliferation, and co-option of transposon-encoded proteins to provide novel cellular functions in higher eukaryotic genomes.

Previous studies have implicated downstream insertion of TEs into functional genes as a major mechanism for TE rescue (in the absence of an incoming promoter element), co-option and the formation of chimeric proteins of novel function. There are numerous examples of such insertions in mammalian genomes. However, our analysis suggests that a more common mechanism of genomic TE co-option involves insertion of an incoming TE upstream of a host gene, oriented in an antisense direction, to form novel bidirectional gene pairs. Further, this appears to occur primarily at genes regulated by CpG-containing promoters, with expression and maintenance of TE integrity facilitated by the inherent bidirectional transcriptional capacity of CpG promoter regions.

### Permissive chromatin environment for insertion and transcription of transposable elements

The majority of CpG islands within mammalian genomes exist in an unmethylated state and are associated with constitutively active genes [17,18]. This state is associated with an open chromatin structure that is thought to render the underlying DNA accessible to various chromatin associated proteins involved in the transcriptional process. We postulated that the majority of CpG islands within mammalian genomes show inherent bidirectional capacity, which coupled with their open chromatin state, make them ideal target sites for the integration and ectopic transcription of opportunistic parasitic transposable elements. In support of this supposition, our analysis of co-opted TEs and rescued gene-trap cassettes revealed that all such insertion events occurred within the vicinity of CpG island regulatory regions. Additional support comes from the use of a synthetic DNA transposon, Sleeping Beauty, as a mutagenesis tool in mammalian genomes. From over 1,000 insertion events mapped in human and mouse cell lines, a small but significant insertion bias towards the 5' upstream regions of genes has been reported [32].

CAGE tag and other expression analyses have shown that CpG island promoters display a broad and complex distribution of transcription start sites when compared to non-CpG island promoters [33,34] possibly contributing to the observed inherent bidirectional potential. Transcription factors like Sp1 can bind at multiple sites and protect the CpG island promoter from being silenced [35-37]. In contrast, when a TE inserts into a non-CpG island or inactive chromatin region, then it is usually silenced via the cis-spreading of silencing factors and/or the homology-dependent RNAi silencing machinery thereby protecting the host genome from the mutagenic potential of subsequent TE insertions [4,5].

## Conclusion

In this study we have described a major molecular mechanism for co-option of transposable elements by host mammalian genomes involving the antisense insertion upstream of inherently bidirectional promoters. This does not affect the integrity of the ancestral gene partner unlike the related gene fusion events that involve downstream DNA insertion into existing genes in the same orientation [1,9]. The net effect of the events we have identified is the acquisition of an additional (and often novel) gene, predicted to acquire a specific gene expression profile dependent of the nature of the associated CpG-containing promoter. Often, the resulting tissue specificity of expression from the incoming DNA element is correlated with its bidirectional partner gene. In many instances such genes carry out essential functions within host organisms and have therefore been 'domesticated' by the host during evolution [7,8]. This appears to be frequently associated with the divergence of specific mammalian lineages during evolution. Further studies on the role of co-opted TEs are warranted to determine the exact roles each plays in mammalian development and speciation.

## Methods

### Selection criteria for the inclusion of co-opted protein-coding genes in this study

Human and mouse genes were selected as co-opted using the following criteria. 1) At least 30% of the coding region was derived from a known transposable element. Transposable element similarity was determined using PSI-BLAST with up to four iterations of the NCBI non-redundant protein database, or direct searches of the Repbase repetitive DNA database [38]. To avoid false positives, simple repeat sequences were filtered out. 2) The gene showed evidence of expression by EST/mRNA cDNA clones from human and mouse UniGene datasets, build #216 and #176, respectively. In addition, we used the Novartis human and mouse tissue gene expression atlas data sets to confirm the cloned EST/mRNA results [27]. 3) We limited the classification to protein-coding genes which were manually annotated and reviewed by RefSeq or UniProt database curators. Mean and median cDNA lengths calculated for bidirectional gene pairs and genome wide genes were retrieved from the UCSC Genome Browser website [39] using the GenBank accession numbers provided in a previous study of bidirectional gene pairs [13]. The whole genome survey of co-opted TEs was limited to human and mouse genomes since both organisms have extensive expression datasets and almost completely sequenced genomes.

### Definition of a bidirectional gene

A gene pair was classified as bidirectional if the consensus transcription start sites were within 1 kb of each other. The major start site peak position was extracted from the CAGE data sets for human hg17 and mouse mm5 genome assemblies [40].

### Detecting the presence or absence of co-opted genes in vertebrate genomes

To determine that a co-opted gene was either present or absent in genomes other than human we used the chained alignments available at the UCSC Genome Brower (human hg18 and mouse mm9 assemblies) [39]. The co-opted gene plus at least 5 kb of flanking sequence were used to assess whether it was present or absent in the compared genome. Furthermore, the flanking 5 kb sequence was searched against the genome sequence datasets to ensure that the TE insertion had not arisen due to segmental duplication.

### Statistical analyses

Chi-squared analysis was used to test the association of co-opted genes with bidirectional promoters. Pearsons correlation coefficient was used to determine the level of correlation between human bidirectional gene pairs from expression data in 79 human tissues [27]. Expression was called as present (P) or absent (A) according to the Affymetrix array analysis tools.

### CpG island analysis

CpG islands were detected according to the criteria set by the UCSC genome browser which is a modification of [14]. CpG islands were predicted by searching the sequence one base at a time, scoring each dinucleotide (+17 for CG and -1 for others) and identifying maximally scoring segments. Each segment was then evaluated for the following criteria: GC content of 50% or greater, length greater than 200 bp, ratio greater than 0.6 of observed number of CG dinucleotides to the expected number on the basis of the number of Gs and Cs in the region of interest.

### PCR and sequence analyses

PCR amplification across the junction regions of the TIGD1 and 4632419I22Rik co-opted genes was performed using HotStar Taq polymerase (Qiagen) using the manufacturer's instructions. Primate genomic DNA panel and the rodent DNA panels were obtained from, Coriell Cell Repositories and Dr Francois Catzeflis, respectively. Primer pairs for the presence or absence of the TIGD1 gene were; TIGD1-f1 5'-CAGGGCTGCCACAAACCC-3' and EIF4-r1 5'-GTCGAACTTGTTGTTCATCCTC-3' or CHRNG-f1 5'-AGCA(A/G)GTTCATTT(T/C)ATTTACTCC-3' and EIF4-r1, respectively. Primer pairs for the presence or absence of the 4632419I22Rik gene were; Brip1-f 5'-AATTCGCGCCTCCCGC-3' and 463Rik-r 5'-GCGTCCTCCAGGACTCTTCG-3', and Brip1-f and Ints2-r 5'-GGAAATTGTACTTCTTGGCAAGG-3', respectively. PCR product integrity was verified by either direct DNA sequencing, or ligating into pGEM-Teasy vector (Promega) followed by sequencing.

## List of abbreviations

TE: transposable element; IGTC: International Gene Trap Consortium; TU: transcriptional unit; CAGE: cap analysis gene expression; EST: expressed sequence tag; LTR: long terminal repeat.

## Authors' contributions

PK and RS designed and performed research, analysed and wrote the paper.

## Supplementary Material

Additional file 1 **Human domesticated genes**. Detailed survey of transposon element derived protein-coding genes in the human genome. Click here for file

Additional file 2 **Mouse domesticated genes**. Detailed survey of transposon element derived protein-coding genes in the mouse genome. Click here for file

Additional file 3 **Anti-sense rescue of gene trap cassettes in mouse ES cell lines**. This dataset provides evidence that CpG island promoters have the capacity of rescuing gene trap cassettes in a bidirectional manner. Click here for file

Additional file 4 **Multiple sequence alignment of TIGD1-EIF4E2 junction in primates**. The multiple sequence alignment displays the 5' insertion site of the TIGD1 gene in the primate lineage. Click here for file

Additional file 5 **Multiple sequence alignments of Brip1-4632419I22Rik and Brip1-Ints2 junctions**. The data show the 5' insertion site of the 4632419I22Rik gene before and after molecular domestication. Click here for file

Additional file 6 **Bidirectional retroposed genes**. A survey of the top 20 transcribed retroposed genes in the human genome showed that six of them were orientated in a bidirectional manner. Click here for file

Additional file 7 **Expression microarray correlation analysis of human TE-derived bidirectional gene pairs**. The data show that a high proportion of human TE-derived bidirectional gene pairs exhibit concordant tissue expression with the neighbouring gene. Click here for file
