# Supplementary material for: Inherent promoter bidirectionality facilitates maintenance of sequence integrity and transcription of parasitic DNA in mammalian genomes
Source: BMC Genomics. 2009 Oct 27;10:498. doi: 10.1186/1471-2164-10-498 (PMC2777200; doi:10.1186/1471-2164-10-498)
Supplement: Additional file 4 — Multiple sequence alignment of TIGD1-EIF4E2 junction in primates. The multiple sequence alignment displays the 5' insertion site of the TIGD1 gene in the primate lineage. [file 1471-2164-10-498-S4.DOC]

Multiple sequence alignment of TIGD1-EIF4E2 junction in primates

Lag.lag TCGATTTGTTTTTAGAAAGCCCTATCTGCGAAGCGCAACAAAAGGAGGGATGCCA----- 55

Ate.geo TCGATTTGTTTTTAGAAAGCCCTATCTGCGAAGCGCAATAAAACGAGGGATGCCA----- 55

Sag.lab TCAATTTGTTTTTAGAAAGCCCTAACTGCAAAGCGCAATAAAACGAGGAATGCCA----- 55

Pan.tro TCCATTTGTTTTCAGAAAGCCCTATCTGCGAAGCGTAATAAAACGAGGTATGCCA----- 55

Pan.pan TCCATTTGTTTTCAGAAAGCCCTATCTGCGAAGCGTAATAAAACGAGGTATGCCA----- 55

Gor.gor TCCATTTGTTTTCAGAAAGCCCTATCTGCGAAGCGTAATAAAACGAGGTATGCCA----- 55

Hom.sap TCCATTTGTTTTCAGAATGCCCTATCTGCGAAGCGTAATAAAACGAGGTATGCCA----- 55

Pon.pyg TCCATGTGTCTTCAGAAAGCCCTATCTGCGAAGCGTAATAAAACGAGGTATGCCA----- 55

Mac.nem TCAATTTGTTTTTAGAAAGTCCTATCTGCGAAGCGCAATAAAACGAGGTATGCCA----- 55

Mac.mul TCAATTTGTTTTTAGAAAGTCCTATCTGCGAAGCGCAATAAAACGAGGTATGCCA----- 55

Lem.cat TCAATTTGCAA--AAAACGCGGTATCTGCGAAGC--AATAAAATGACGTGTGCCAGTACT 56

Oto.gar TCAATTTGTAA--AAAACGCAGTGTCAGCGAAGC--AATAAAACGAGGTATGCCAGGACG 56

** ** ** * ** * * * ** **** ** **** ** * *****

Lag.lag -TACTAGAGTGGCGCCGCC----GCCAAAGAAAAGCCTCGGGAAGGTTAAGAGAGATGCC 110

Ate.geo -TACTAGAGTGGCGCCGCC----GCCAAAGAAAAGCCTAGGGAAGGTTAAGAGAGATGCC 110

Sag.lab -TACTAGAGTGGCGCCGCC----GCCAAAGGAAAGCCTCGGGGAGGTTAACAGAGATGCC 110

Pan.tro -TAGTACAGTGGCGCCCCC--CCGACAAAGGAAAGCCTCCGGGAGGTCAAGAGAGATACC 112

Pan.pan -TAGTACAGTGGCGCCCCC--CCGACAAAGGAAAGCCTCCGGGAGGTCAAGAGAGATACC 112

Gor.gor -TAGTACAGTGGCGCCCCG--CCGACAAAGGAAAGCCTCCGGGAGGTCAAGAGAGATACC 112

Hom.sap -TAGTACAGTGGCGCCGCGA-CCGACAAAGGAAAGCCTCCGGGAGGTCAAAAGAGATACC 113

Pon.pyg -TAGTACAGTGGCGGGCCA--CCGACAAAGGAAAGCCTGGGGGAGGTCAAGAGAGATACC 112

Mac.nem -TAGTATAGTGGCGCTGCC----GCCAAAGGAAAGCCTCGGGAAGGTTAAGAGAGATACC 110

Mac.mul -TAGTATAGTGGCGCTGCC----GCCAAAGGAAAGCCTCGGGAAGGTTAAGAGAGATACC 110

Lem.cat ACAGTGTAGTAGCGTCACTGCCAGACAGGAAAGAGCCTGGAAGAGGTTGAGAGAGATACC 116

Oto.gar GTAGTACAGTAGCGACGCTGAGAGAGCGAAAGGAGCCTCCAAGAGGTTAAGAG--ATGCC 114

* * *** *** * * ***** **** * ** ** **

Lag.lag CGAGGGTGCGTCCCGCACTGAAGGAGGAGGAGGAGG------GTTTTTACCAAACAGCGA 164

Ate.geo CAAGGGTGCCTCCCGCACTGGAGGAGGAGGAGGAGGAGGCGGGTTTTTACCAAGCAGCGA 170

Sag.lab GGAGGGTGCGTCCCGCACTGGAGGAGGAGGAGG---------GTTTTTACCAAGCAGCGA 161

Pan.tro AGAGAGTGCGTCCCGCACTGGAGGAAGAGGAAG---------GTTTTTACCAAGCAGCGA 163

Pan.pan AGAGAGTGCGTCCCGCACTGGAGGAAGAGGAAG---------GTTTTTACCAAGCAGCGA 163

Gor.gor AGAGAGTGCGTCCCGCACTGGAGGAAGAGGAAG---------GTTTTTACCAAGCAGCGA 163

Hom.sap AGAGAGTGCGTCCCGCACTGGAGGAAGAGGAAG---------GTTTTTACCAAGCAGCGA 164

Pon.pyg GCAGAGTGCGTCCCGCACTGGAGGAAGAGGAAG---------GTTTTTACCAAGCAGCGA 163

Mac.nem GGAGAGTGCGTCCCGCACTGGAGGAGGAGGAGG---------GTTTTTACCAGGCAGCGA 161

Mac.mul GGAGAGTGCGTCCGGCACTGGAGGAGGAGGAGG---------GTTTTTACCAGGCAGCGA 161

Lem.cat GGAGGGTGCGGCCAGCACTGGAGGTGGAGTCGGG--------ATTTTTACCAAGCTGCGA 168

Oto.gar AGAGGGTGAGGCC-GTACTGGAGTTGGAGTTGGG--------ATTTTTACCAAGCAGCGA 165

** *** ** * **** ** *** * ********* * ****

Lag.lag CTCCAGAGCCCGCGCCGTCAACGACCACGGTGCTGCCTT-TTTTCCGAGCCGCTGCGGGA 223

Ate.geo GTCCAGAGCCCGCGCCGTCAACGACCACGGTGCTGCCTT-TTTTCCGAGCCGCTGCGGGA 229

Sag.lab GTCCAGAGTCCGCGCCGTCAACGACCGCGGTGCTGTCTT-TTTTCCGAGCCGCTGAGGGA 220

Pan.tro GTCCAGAGCCCGCGCTGTCAACGAC-GCGGTGCTGCCTT-TTTTCCGAGCCGCTGCGGGA 221

Pan.pan GTCCAGAGCCCGCGCTGTCAACGAC-GCGGTGCTGCCTT-TTTTCCGAGCCGCTGCGGGA 221

Gor.gor GTCCAGAGCCCGCGCTGTCAACGAC-GCGGTGCTGCCTT-TTTTCCGAGCCGCTGCGGGA 221

Hom.sap GTCCAGAGCCCGCGCCGTCAACGAC-GCGGTGCTGCCTT-TTTTCCGAGCCGCTGCGGGA 222

Pon.pyg GTCCAGAGCCCGCGCTGTCAACGAC-GTGGTGCTGCCTT-TTTTCCGAGCCGCTGCGGGA 221

Mac.nem GTCCAGAGCCCGCGCTGTCAACGAC-GCGGTGCTGCCTT-TTTTCGGAGGCGCTGCAGGA 219

Mac.mul GTCCAGAGCCCGCGCTGTCAACGAC-GCGGTGCTGCCTT-TTTTCGGAGGCGCTGCAGGA 219

Lem.cat GTCCAGAGCCCGCGCTGTCAACGAC-GCGGTGCTGCCTTGTTTTCCGAGCCGCTGCGGGA 227

Oto.gar GTCCAGAGCCCGCGCTGTCAACGAC-GCCGTGCTGCTTTCTTTTCTGAGCCGCGGCGGGA 224

******* ****** ********* ****** ** ***** *** *** * ***

Lag.lag GGGTGCCAGGACACGCTCCCTTAGGAGAACTGGCGGCTCACAAACACT-TGGACAGGGAA 282

Ate.geo GGGTGC-AGGGCACGCTCCCTTAGGAGAACCGGCGGCTCACAAACACT-TGGACAGGGAA 287

Sag.lab GGGTGCCAGGACATGCTCCCTTAGGAGAACTGGCGGCTCACAAACACC-TGGACTGGTAA 279

Pan.tro GGGGGCCAGGACACGCTCCCTTAGGAGAGCGGGCGGGTCACAAGGACC-TGGACAGAGAC 280

Pan.pan GGGGGCCAGGACACGCTCCCTTAGGAGAGCGGGCGGGTCACAAGGACC-TGGACAGAGAC 280

Gor.gor GGGGGCCAGGACACGCTCCCTTAGGGGAGCGGGCGGGTCACAAGGACC-TGGACAGAGAC 280

Hom.sap GGGGGCCAGGACACGCTCCCTTAGGAGAGCGGGCGGGTCACAAGGACC-TGGACAGAGGC 281

Pon.pyg GGGGGCCAGGACACGCTCCCTTAGGAGAGCCGGCGGGTCACAAGGACC-TGGACAGAGAC 280

Mac.nem GGGCGCCTGGACACGCTCCCTTAGGAGAGCGGGCAGGTCACA-GGACC-TGGACGGCGAC 277

Mac.mul GGGCGCCTGGACACGCTCCCTTAGGAGAGCGGGCAGGTCACA-GGACC-TGGACGGCGAC 277

Lem.cat GGG-GACGAGACGCGCTCCCTTAAGCG-GCCGGCGACCCACACAGACGACGAACCTGGAC 285

Oto.gar GGG-GCCAGCGCGCGCTCCCTTAGGCG-GCGGGCGACCCACAGGGTTCAGGAACCAGAAC 282

*** * * ********* * * * *** **** * **

Lag.lag AGAGCTGAGGCCAGCAGCCAGCTCCACCGCTGAGGCCAGCCCTGTGCGCAGCTGCCTGGG 342

Ate.geo AGAGCTGAGGCAAGCAGCCAACTCCGCCGCTGAGGCCAGCCCTCTGCGCAGCTGCCTGGG 347

Sag.lab AGACCTGAAGCCAGCAGCCAACTCCGCCGCTGAGGCCAGCTCTCTGCGCAGCCGCCCCGG 339

Pan.tro AGAACTGAGGCCAGCAGCCAGCTCCGCCGCTGAGTCCAGCCCTCTGCGCAGCCGCCCTGG 340

Pan.pan AGAACTGAGGCCAGCAGCCAGCTCCGCCGCTGAGTCCAGCCCTCTGCGCAGCCGCCCTGG 340

Gor.gor AGAACTGAGGCCAGCAGCCAGCTCCGCCGCTGAGTCCAGCCCTCTGCGCAGCCGCCCTGG 340

Hom.sap AGAACTGAGGCCAGCAGCCAGCTCCGCCGCTGAGTCCAGCCCTCTGCGCAGCCGCCCTGA 341

Pon.pyg AGAGCTGAGGCCAGCAGCCAGCTCCGCCGCTGAGGCCAGCCCTCTGCGCAGCCGCCCTGG 340

Mac.nem AAAGCTGAGACCTGAAGCCAGATCCGCCGCTGAGGCCAACCCTCTGCGCAGCCGCCCTGG 337

Mac.mul AAAGCTGAGACCTGAAGCCAGCTCCGCCGCTGAGGCCAACCCTCTGCGCAGCCGCCCTGG 337

Lem.cat AGAGCCGAGGCCCGCAG-CTACCTAGCCATTGCGACCAGGCCTCTGCGCAGCCGCCCTGG 344

Oto.gar ACAGCCGAGAACAGC---CAGCCCAGCCACTGAGTCTAAGTCTCCGCGCAGCTGCTCTGG 339

* * * ** * * ** ** * * * ** ******* ** *

Lag.lag GCTGGCGTTCTGGGAT-AGCAGCCCCCAG-CTGGGCCTGCGCGCGGACGTTCCGGGGCGC 400

Ate.geo GCTGGCGTCCCGGGAT-AGCAGCCCCCAA-CCGGGCCTGCGCGCGGACGTTCCGGGGCGC 405

Sag.lab GCTGGCGTCCCGGGAT-AGCAGCCCCCAG-TCGGGCCTGCGCGCCGACGTTCCGGTGCGC 397

Pan.tro GCTGGCGTCCCGAGAT-AGCCGCTTCCAAACCGGGCCTGCGCGCCGACGTTCCGCTGCGC 399

Pan.pan GCTGGCGTCCCGAGAT-AGCCGCTTCCAAACCGGGCCTGCGCGCCGACGTTCCGCTGCGC 399

Gor.gor GCTGGCGTCCCGAGAT-AGCCGCTTCCAAACCGGGCCTGCGCGCCGACGTTCCGCTGCGC 399

Hom.sap GCTGGCGTCCCGAGAT-AGCCGCTTCCAAACCGGGCCTGCGCGCCGACGTTCCGCTGCGC 400

Pon.pyg GCTGGCGTCCCAAGAT-AGCCGCTCCCAAACCGGGCCTGCGCGCCGACGTTCCGCTGCGC 399

Mac.nem GTTGGCGTCCCGAGAT-AGACGCCCCCAAACCGGGCCTGCGCGTGGACGTTCCGCTGTGC 396

Mac.mul GCTGGCGTCCCGAGAT-AGACGCCCCCAAACCGGGCCTGCGCGTGGACGTTCCGCTGTGC 396

Lem.cat GCTGGCGCCCTGGGATTAGCTGCCCCCCACCG-GGCATGCGCGCCGACGTTCCGCCGCGC 403

Oto.gar GCTGGTGTCCTAGGATTAGCTGCCCCCCACCGAGGCCTGCGCACAGACGTTCCGCTGCGT 399

* *** * * *** ** ** ** *** ***** ********* * *

Lag.lag CTCGCGCAAAACCGGAAGTACCCGGGCCTAAGGCTGAGGGACCCGGTGGAGCGGAAGTCA 460

Ate.geo CTCGCGCAAAACCGGAAGTACCCGGGCCTAAGGCTGAGGGACCCGGTGGAGCGGAAGTCA 465

Sag.lab CTCGCGCAAAACCGGAAGTAGCCGGGCCTAAGGCTGAGGGACCCGGTGGAGCGGAAGTCA 457

Pan.tro CCCGCGCAAAACCGGAAGTACCCGGGCCCAAGGCTGAGGGACCCGGTGGAGCGGAAGTCA 459

Pan.pan CCCGCGCAAAACCGGAAGTACCCGGGCCCAAGGCTGAGGGACCCGGTGGAGCGGAAGTCA 459

Gor.gor CCCGCGCAAAACCGGAAGTACCCGGTCCCAAGGCTGAGGGACCCGGTGGAGCGGAAGTCA 459

Hom.sap CCCGCGCAAAACCGGAAGTACCCGGGCCCAAGGCTGAGGGACCCGGTGGAGCGGAAGTCA 460

Pon.pyg CCCGCGCAAAACCGGAAGTACCCGGGCCCAAGGCTGAGGGACTCGGTGGAGCGGAAGTCA 459

Mac.nem CCCGCGCAAAACCGGAAGTACCCGGGCCTAAGGCTGAGGGACCCGGTGGAGCGGAAGTCA 456

Mac.mul CCCGCGCAAAACCGGAAGTACCCGGGCCTAAGGCTGAGGGACCCGGTGGAGCGGAAGTCA 456

Lem.cat CCCGCGCA--ACCGGAAGTGGCCGGGCCCAAGGCGGAGGGACCCCGTGGAGCGGAAGTCA 461

Oto.gar CCCGCGCA--ACCGGAAGTGACCGGGCCTGAGGCGGAGGGACCAGGTGGAGCGGAAGTCA 457

* ****** ********* **** ** **** ******* ***************

Lag.lag CTCCTTGAGGCAGTGGCGACAGCGGCGGCGA 491

Ate.geo CTCCTTGAGGCAGTGGCGACAGCGGCGGCGA 496

Sag.lab CTCCTTGAGGCAGTGGCGACAGCGGCGGCGA 488

Pan.tro CTCCCTGAGGCAGTGGCGACAGCGGCGGCGA 490

Pan.pan CCCCCTGAGGCAGTGGCGACAGCGGCGGCGA 490

Gor.gor CTCCCTGAGGCAGTGGCGACAGCGGCGGCGA 490

Hom.sap CTCCCTGAGGCAGTGGCGACAGCGGCGGCGA 491

Pon.pyg CTCCCTGAGGCAGTGGCGACAGCGCCGGCGA 490

Mac.nem CTCCCTGAGGCAGTGGCGACAGCCGCGGCGA 487

Mac.mul CTCCCTGAGGCAGTGGCGACAGCCGCGGCGA 487

Lem.cat CTCCGTGAGGCTGTGGAGACAGCGGCAGCGA 492

Oto.gar CTCCGTGAGGCAGTGGCGACAGCGGCGGCGA 488

* ** ****** **** ****** * ****

Species used in the multiple alignment.

Hom.sap - Homo sapiens, Pan.tro - Pan troglodytes, Pan.pan - Pan paniscus, Gor.gor - Gorilla gorilla, Pon.pyg - Pongo pygmaeus, Mac.mul - Macaca mulata, Mac.nem - Macaca nemestrina, Sag.lab - Saguinus labiatus, Ate.geo - Ateles geoffroyi, Lag.lag - Laothrix lagotricha, Lem.cat - Lemur catta, and Oto.gar - Otolemur garnettii

The 5’UTR of the tigger-derived gene, TIGD1, the putative terminal inverted repeat and the 5’UTR region of the EIF4E2 gene are shown in blue, green and red, respectively.
