# Supplementary material for: Inherent promoter bidirectionality facilitates maintenance of sequence integrity and transcription of parasitic DNA in mammalian genomes
Source: BMC Genomics. 2009 Oct 27;10:498. doi: 10.1186/1471-2164-10-498 (PMC2777200; doi:10.1186/1471-2164-10-498)
Supplement: Additional file 5 — Multiple sequence alignments of Brip1-4632419I22Rik and Brip1-Ints2 junctions. The data show the 5' insertion site of the 4632419I22Rik gene before and after molecular domestication. [file 1471-2164-10-498-S5.DOC]

Multiple sequence alignments of Brip1-4632419I22Rik and Brip1-Ints2 junctions

Figure S2A Multiple sequence alignment of Brip1-4632419I22Rik junction

M_musC57 CCCTCGGTTCCCAGTCAGCCAATCACACAGCCAGACCGGAAAAGGAAACAGCCAATCATC 60

M_m-cast CCCTCGGTTCCCAATCAACCAATCACACAGCCACACCGGAAAAGGAAACAGCCAATCATC 60

M_spre CCCTCGGTTCCCAATCAGCCAATCACACAGCCAGACCGGAAAAGGAAACAGCCAATCATC 60

M_caro CCCTCGGTTCCCAATCAGCCAATCACACAGCCAGGCCGGAAAAGGAAACAGCCAATCATC 60

M_famu CCTTCGGTTCCCAATCAGCCAATCACACAGCCAGGACGGAAAAGGAAACAGCCAATCATC 60

M_saxi CCCTCGGTTCCAAATCAGCCAATCACACAGCCAGGCCGGAAAAGTGAACAGCCAATCATC 60

M_paha CCCTCGGTTCCCAGTCAGCCAATCACACAGTCAGGCCGGAAAAGGAAACAGCCAATCATC 60

** ******** * *** ************ ** ******** **************

M_musC57 GTACGAGGCCAACGCTATACCGCCCAATGGGAGCCCGCGAAGTAAAATCAACCCTCGAGT 120

M_m-cast GTACGAGGCCAACGCTGTACCGCCCAATGGGAGCCCGCGAAGTAAAATCAACCCTCAAGT 120

M_spre GTACGAGGCCAACGCTGTACCGCCCAATGGGAGCCCGCGAAGTAAAATCAACCCTCGAGT 120

M_caro GTACGAGACCAACGCTGTACCGCCCAATGGGAGTCCGCGAAGTAAAATCAACCCCCAAGT 120

M_famu GTACGAGTCCAACGCTGTACCGCCCAATGGGAGCCCGCGAAATAAAATCAACCCTCAAGT 120

M_saxi GTACGAGGCCTACGCTGTACCGCCCAATGGGAGCCCGCGAAGTAAAATCAACCCTAAAGT 120

M_paha GTACGAGGCCTACGCGGTACCGCCCAATGGGAGCCCGCGAAGTAAAATCAACCCTAAAGT 120

******* ** **** **************** ******* ************ ***

M_musC57 ACTTGGCGGGACCGGAAGACGAGCACCGCCCCTCCCCCAAACCCCACCCCGCGAAAAAGA 180

M_m-cast ACTTGGCGGCACCGGAAGACGAGCACCGCCCCTCCCCCAAACCCCACCCCGCGAAAAAGA 180

M_spre ACTTGGCGGCACCGGAAGACGAGCACCGCCCCTCCCCCAAACCCCACCCCGCGAAAAAGA 180

M_caro ACTTGGCGGCACCGGAAGATGAGCACCGCCCCTCCCCAACCCCCCACCCCGCGAGAAAGA 180

M_famu ACTTGGCGGCACCGGAAGACGAGCACCGCCCCTCCCCCAAACCCCACCCCGCGGGAAAGA 180

M_saxi ACTTGGCAGCACCGGAAGACAAGCAC--------------------CCCCGCGAGAAAGA 160

M_paha CCTTGGCGGCACCGGAAGACGAGCAC--------------------CCCAGCGAGAAAGA 160

****** * ********* ***** *** *** *****

M_musC57 CTGGGACA--AGTTCCGCACATTTGCTTGAAGGA--AAAAAAAAAA-------TTCTTGT 229

M_m-cast CCGGGACA--AGTTCCGCACATTCGCTTGAAGGAG-AAAAAAAAAA-------TCCTTGT 230

M_spre CCGGGACA--AGTTCCGCACATTTGCTTGAAGGAAAAAAAAAAAAA-------TTCTTGT 231

M_caro CCGGGGCA--AATTCCGCACATTTGCTTGAAGGGAAAAAAAAAA---------TTCTTGT 229

M_famu CCGGGACA--AATTCCGCACATTTGCTTGAAGGGAAAAAAAAAAAAAA-----TTCTTGT 233

M_saxi CCGGGACA--AATTCCGCACGTTTGCTTGAAGGAAGCAAAAAAAAAA------AAAAAAT 212

M_paha CCCGGACACAAATTCCGCACGTTTGCTTGAAGGAAGAAAAAAAAAAMAAAAAAATCTTGT 220

* ** ** * ******** ** ********* ******* *

M_musC57 ACTGTGAGACTTTAAGAAGCACAAAATGGGCGTTAGACGGGTCTTACATTTGGTGCATTG 289

M_m-cast ACTGTGAGACTTTAAGAAGCACAAAATGGGCGTTAGACGGGTCTTACATTTGGTGCATTG 290

M_spre ACTGTGAGACTTTAAGWAACACAAAATGGGCGTTAGACGGGTCTTACATTTGGTGCATTG 291

M_caro ACTGTGAGACTTTAAGAAGCACAAAATGGCCGTTAGACGGGTCTTRCATTTGGTGCATTG 289

M_famu ACTGTGAGACTTTAAGAAGCACAAAATGGCCGTTAGACGGGTCTTACATTTGGTGCATTG 293

M_saxi CTTGTGGGACTTTAAGAGGCACAAAATGGCCGTTAGACAGGTCTTACATTTGGTGCATTG 272

M_paha ACTGTTGGACTTTAAGAAGCACAAAATGGCCGTTAGACGGGTCTTACATTGGGTGCATTG 280

*** ********* ********** ******** ****** **** *********

M_musC57 ACCGGGAATCGTCCTGCTGGCGGGTCCAGTGACAGGAGACCGGAGGATTGTTCGAGCAGG 349

M_m-cast ACCGGGAATCGTCCTGCTGGCGGGGCCAGTGACAGGAGACCGGAGGATTGTTCGAGCAGG 350

M_spre ACCGGGAATCGTCCTGCTGGCGGGGCCAGTGACAGGAGACCGGAGGATTGTTCGAGCAGG 351

M_caro ACCGGGAATCGTCCTGCTGGCGGGGCCAGTGACAGGAGACCCGAGGATTGTTCGAGCAGG 349

M_famu ACCGGGAATCGTCCTGCTGGCGGGGCCAGTGACAGTAGACCGGAGGATTGTTCGAGCAGG 353

M_saxi ACCGGGAATCGTCCTGCTCGCGGGGCGAGTGACAGGAGACCGGAGGATTGCTCGAGCAGG 332

M_paha ACCGGGAATCGTGCTGCTCGGCGGGCCAGTGACAGGAGACCGGAGGATCGCTAGACCAGG 340

************ ***** * ** * ******** ***** ****** * * ** ****

M_musC57 TACCGCGGCTGTGTCTCTTTGTGTCCGCCCTTGGCGTGCGTCTATTCCGCGCGTCTATTC 409

M_m-cast TACCGCGGCTGTGTCTCTTTGTGTCCGCCCTTGGCGTGCGTCTATTCCGCGCGTCTATTC 410

M_spre TACCGCGGCTGTGTCTCTTTGTGTCCGCCCTTGGCGTGCGTCTATTCCGCGCGTCTATTC 411

M_caro TACCGCGGCTGTGTCTCTTTGTGTCTGCCCTCGGCGTGCGTCTGTTCCGCGCGTCTATTC 409

M_famu TACCGCGGCTGTGTCTCTTTGTGTCTGCCCTTGGCGTGCGTCTATTCCGCGCGTCCATTC 413

M_saxi TACCGCGGCTGTGTCCCTCTGTGTCTGCCCTTTGCGTGAGTCTAGTCCGCGCGTCTATTC 392

M_paha TACCGCGGCTGTGTCTCTTTGTGTCTGCCCTTTGCGTGCGTCTG-------------TTC 387

*************** ** ****** ***** ***** **** ***

M_musC57 CGTCT---------GTTCCCTCTATTTTGAGTCGGCTGCAAGTCAGTTTGGTTTCGGTTT 460

M_m-cast CGTCTATTCCGTCTGTTCCCTCTATTTTGAGTCGGCTGCAAGTCAGTTTGGTTTCGGTTT 470

M_spre CGTCT---------GTTCCCTCTATTTTGAGTCGGCTGCAAGTCAGTTTGGTTTCGGTTT 462

M_caro CGTCT---------GTTCCCTCTATTTTGGGTCGGCTGCAAGTCAGTTTGGTTTCGGTTT 460

M_famu CGTCT---------GTTCCCTCTATTTTGAGTCGGCTGCAAGTCAATTTGGTTTCGGTTT 464

M_saxi CGTCT---------GTTCCCTCTATTTTGAGTCGGCTGCAAGTCAGTTTGGTTTCGGTTT 443

M_paha CGTCT---------GTTCTCTCTATCTTGAGTCGGCTGCAAGTCAGTTTGGTTTCGGTTT 438

***** **** ****** *** *************** **************

M_musC57 GCAA--------AGGAGTTGTCTGCCGGGGGTTGCCAGAAGCGAC------------CTT 500

M_m-cast GCAA--------AGGAGTTGTCTGCCGGGGGTTGCCAGAAGCGAC------------CTT 510

M_spre GCAACTCGACCAAGGAGTTGTCTGCCAGTGGTTGCCAGAAGCGGC------------CTT 510

M_caro GCAACTCGACCAAGGAGTTGTCTGCCGGGGGTTGCCAGAAGCGGC------------CTC 508

M_famu GCAACTCGACCAAAGAGTTGTCTGCCGGGGGYTGCCAGAAGCGGC------------CTT 512

M_saxi GCAACTCGACCAAGGAGTTGTCTGCTGGGGGTTGCCAGAAGCGGC------------CTT 491

M_paha GCRACTCAACCAAGGAGTTGTCTGCCGTGGGTAGGCAGAAGCGGCGGGTAGACGTGCTTT 498

** * * *********** ** * ******** * *

M_musC57 AACGCTGCGGTAA---- 513

M_m-cast AACGCTGCGGTAA---- 523

M_spre AACGCCGCGATAA---- 523

M_caro AACGCCGCGGTAA---- 521

M_famu AACGCCGCGGTAA---- 525

M_saxi AACGCCGCGGCAGGCAA 508

M_paha AACGCCGCGACAA---- 511

***** *** *

M_musC57 - Mus musculus C57BL/6, M_m-cast - Mus musculus castaneus, M_spre - Mus spretus, M_caro - Mus caroli, M_famu - Mus famulus, M_saxi - Mus saxicola, and M_paha - Mus pahari

Red nucleotides denote the target site duplication and blue nucleotide are the endogenous retrovirus-derived gene, 4632419I22Rik.

Figure S2B Multiple sequence alignment of Brip1-Ints2 junction

R_ratt ---CCCGGTTCTCAGTCAGCCAATCACACAGTCAGGCCAAAA-GGTAAACAGCCAATCAT 56

R_norv ---CCCGGTTCTCAGTCAGCCAATCACACAGTCAGGCCAAAA-GGTAAACAGCCAACCAT 56

S_muel ---CCCGGTTCTCAGTCAGCCAATCACACAGTCAGGCCAAAA-GGTAAACAGCCAATCAT 56

L_saba ---CCCGGTCYCCAGTCAGCCAATCACACAATCAGGCCAAAA-AGGAAACAGCCAATCAT 56

N_rapi CTCCCCGGTCCCCAGTCAGCCAATCACACAATCAGGCCAAAA-AGGAAACAGCCAATCAT 59

A_flav --CCTCTGTTCCCAATCAGCCAATCACACAGCCAGGCCGGAACATTAA-CAGCCAATCAT 57

A_sylv CCCCTCTGTTCCCAAACAGCCAATCACACAGCCAGGCCGGAACATTAA-YAGCCAATCAT 59

M_couc -CCCTCGGTTCCCAGTCAGCCAATCACACAGCCCGGCCAGAAAAGTAAATAGCCAATCAT 59

* * ** ** ************** * **** ** ** ****** ***

R_ratt CGTACGAGGCCAATACTGTACCGCCCAATGGGAGCCTGCGAAATAAAATCAGCCCTAATG 116

R_norv CGAACGAGGCCAATACTGTACCGCCCAATGGGAGCCTGCGAAATAAAATCAACCCTAATG 116

S_muel CGTACGAGGCCGATACTGTACCRCCCAATGGGAGCCTGCGAAATAAAATCAGCCCTAATG 116

L_saba CGTACGAGGCCAATACTGTACCGCCCAATGGAAGACTGCGAAGTAAAATCAGCCCTTATG 116

N_rapi CGTACGAGGCCAATACTGTACCGCCCAATGGGAGTCTGCGAAATAAAATCAGCCCTAATG 119

A_flav CGTACGAGGCTGACACTATGCCGCCCAATGGGRGCCTGCGAAATAAAATCAACCCTAATG 117

A_sylv CGTACGAGGCTGACACTATGCCGCCCAATGGGAGCCTGCGAAATAAAATCAACCCTAATG 119

M_couc CGTACGAGGCCAACGCTGTACCGCCCAATGGGAGCCTGTGAAATAAAATCAACCCTAATG 119

** ******* * ** * ** ******** * *** *** ******** **** ***

R_ratt TACTAGACCGCACCGGAAGGCGGTCAC-------CCCCAGCGAAAAAGACTGGAACAAAT 169

R_norv TACTAGACCGCACCGGAAGGCGATCAC-------CCCCAGAGAGAAAGACTGGAACAAAT 169

S_muel TACTAGACCGCACCGGAAGGCGATCAC-------CCCCAGCGAGAAAGACTGGAACAAAT 169

L_saba TACTAGACCGCRCCGGAAGGCGATCAC-------CCCCAGCGAGAAAGACTGGAACAAAT 169

N_rapi TACTAGACCGCACCGGAAGGCGATCAC-------CCCCGGCGAGAAAAACTGGAACAAAA 172

A_flav TACTTGGTGGCACCGGAAACTGATC--------ACCCCGGCGAGAAAAACTGGAACAAAT 169

A_sylv TACTTGGCGGCACCGGAAACTGATC--------ACCCCCGCGAGAAAAACTGGAACAAAT 171

M_couc TACTTGGCGGCACCGGAAGGCGATCCCTTCCCTACCCCCGCGAGAAAGAC--GTGCAAAT 177

**** * ** ****** * ** **** * ** *** ** * ****

R_ratt TCCGCAGATTTGCTTGAAGGAAAGGAAAAAAGTCTTGTACCACTTTTGGGRACGGATGAT 229

R_norv TCCGCAGATTTGCTTAAAGGAAAGGAAAAAAGTCTTGTACCACTTTTGGGAACGGATGAT 229

S_muel TCCGCAGATTTGCTTGAAGGAAAGGAAAAAAGTCTTGTACCACTTTTGGGAACGGATGAT 229

L_saba TCCGCAGATTTGCTTAAAGGAAAGGGAAAAAGTCTTGTACCACTTTTGGGAACGGATGAT 229

N_rapi TCCGCAGATTTGCTTAAAGGAAAGGAAAAAAGTCTTGTACCACTTTTGGGWACGAATGAT 232

A_flav TCCGCAGATCTGCTTGAAGGAAAC-AAAAAAGTCTTGTACCACTTTTGGAAATGGATGAT 228

A_sylv TCCGCAGATCTGCTTGAAGGAAAC-AAAAAAGTCTTGTACCRCTTTWGGAAAWGGATGAT 230

M_couc TCCGCAGGTTTGCTTGAAGGAAAC-A---AAGTCTTGTACCACTTTTGGAAACCGATGAT 233

******* * ***** ******* ************ **** ** * *****

R_ratt CTAACCTAGACAGCCAGTTCCCTATGACTGGAAGACAATTTTATGACGAAAAGGAAACAT 289

R_norv CTAACCTAGACAGCCAGTTCCCTATGACTGGAAGACAATCTTATGACGAAAAGGAAACAT 289

S_muel CTAACCTAGACAGCCAGTTCCCTATCACTGGAAGACAGTTTTACGACGAAAAGGAAACAT 289

L_saba CTAACCTAGACAGCCAGTTCCCTATGACTGGAAGACAATTTTGCGACGAAAAAGAAACAT 289

N_rapi CTAACCTAGACAGCCAGTTCCCTATGACTGGAAGACAATTTTGCGACGAAAAAGRAACAT 292

A_flav TTAACCTAGACAGCCAGGTCCCTATGGCTGGAAGACTAACTTGCGACGAGAAAGAAACAA 288

A_sylv TTAACCTAGACAGCCAGGTCCCTATGGCTGGAAGACTAACTTGCGACGAAAAAGAAACAA 290

M_couc TTAACCTAGACAGCTAGTTCGCTATGACTGGAAGAGTAATTTCCGACGAAAAGGAAACTY 293

************* ** ** **** ******** ** ***** ** * ***

R_ratt TTTT-CCTGTTGTCTTATCACGCAAATAAAACTTCCCATGGTTACAGTATCCTAAGAAAG 348

R_norv TTTT-CCTGTTGTCTTATCACGCAAATAAAACTTCCCATGGTTACACTA----------A 338

S_muel TTTT-CCTGTTGTCTTATCACACAAATAAAACTTCCCATGGCTACAGTATCCTAAGAAAG 348

L_saba TTTTTCCTGTTGTCTTATCACACAAGTAAAACTTCCCATGGTTACAGTATCCTAAGAAAG 349

N_rapi TTTT-CCTGTTGTCTTATCACACAAGTAAAACTTCCCATGGTTACAGTATCCTAAGAAAG 351

A_flav TTCT-----TTTTCCTGTC--GCAACTAAAATTTCCCATGGTCGCAGTATCCAAAGAAAA 341

A_sylv TTCT-----TTTTCCTGTC--GCAACTAAAATTTCCCATGGTCGCAGTATCCAAAGAAAA 343

M_couc TTTT-----TCCTGTGATCACGCAAGTAAAACTTCCCATGGTTACAGTATCCAAAAAAAA 348

** * * * ** *** ***** ********* ** **

R_ratt G-------AGAGTGCAAACTGCACCAGAATTGTCACTACA-------------------- 381

R_norv G-------AGAGTGCAAACTGCACCAGAATTGTCACTACA-------------------- 371

S_muel G-------AGAGTGCAAACTGCACCAGAATTGTCACTACA-------------------- 381

L_saba G-------AGAACGCAAACTGCACCAGAATTGTCACTACAATGTCAAAATAAGTGAGAAG 402

N_rapi G-------AGAAGGCAAACTGCACCAGA-------------------------------- 372

A_flav A-------GGAACACAAACTGCACCAGAATTGTCACTACA-------------------- 374

A_sylv A-------GGAACACAAACTGCACCAGAATTGTCACCAAA-------------------- 376

M_couc AAACAAGAGGAACGCAAACTGCTCCAGAATTGTCACTACA-------------------- 388

** ******** *****

R_ratt ---------------------------------------ATGCCAGAATAAGTGRGTAGG 402

R_norv ---------------------------------------ATGCCAGAATAAGTGAGTAGG 392

S_muel ---------------------------------------ATGCCAAAATAAGTGAGTAGG 402

L_saba GTCCCATGGCACTTCGAACATTAATTAATTGTCACTACAATATCAAAATAAGTGAGAAGG 462

N_rapi ---------------------------------------ATGTCAAAATAAGTGAGAAGG 393

A_flav ---------------------------------------ATGTCAAAATAAGTGAAAAGG 395

A_sylv ---------------------------------------ATGTCAARATAAGTGAAAAGG 397

M_couc ---------------------------------------ATGCCAAAATAAGAGAGAAAG 409

** ** ***** * * *

R_ratt TCCCATGGCACTTCGAACATTA----TTCCGTGATGTTCAAGGTCT---CGGATAGAATC 455

R_norv TCCCATGGCACTTCGAACATTA----TTCCGTGATGTTCAAGGTCT---CGGATAGAATC 445

S_muel TCCYATGGCACTTCGAACATTA----TTCCGTGATGTTCAAGGTCT---CGGATARAATC 455

L_saba TCCCATGGCACTTCGAACATTAATTATTCCGTGATGTTCAAGATCTTCTCAGATAGAATC 522

N_rapi YCCCATGGCACTTCGAACATTAATTATTCCGTGATGTTCAAGATCTTCTCGGATAGAATC 453

A_flav TCCCGTGGCACTTTGTACGTTA----TTCCGAGATATTCAAGGCTTTCTCGGATAGAAT- 450

A_sylv TCCCGTAGCACTTTGTACGTTA----TTCCGAGATATTCAAGGCTTTCTCGGATAGAAT- 452

M_couc TCCCGTGGCACTTTGTACTTTA----TTCCGTGACATTCAAAGCCTTCTCCGGTAGAATC 465

** * ****** * ** *** ***** ** ***** * * * ** ***

R_ratt TGAGGCGTATAATCTCTCCCGATCACATAGATC----ACTAGGGTTTACCTAACTTACAT 511

R_norv TGAGGCGTATAATCTCTCCCGATCACATAGATC----ACTAGGGTTTACCTAACTTGCAT 501

S_muel TRAGGCGTATAATCTCTCCCGATCACATAGATC----ACTAGGGTTTACCTAACTAACAT 511

L_saba TGAGGCATATAATCTCTCCAGATCACATAGATC----ACTAGGGTTTACCTAACTTACAT 578

N_rapi TGAGGCGTATAATCYCTCCAGATCACATAGATC----ACTAGGGTTTACCTAACTTGCAT 509

A_flav -GAGGCGGATAATCTCTCCAGATCACATCGACCTAACACTAGGGTTTACCTACCTTACAT 509

A_sylv -GAGGCGGATAATCTCTCCAGATCACATCGACCTAACACTAGGGTTTACCTACCTTACAT 511

M_couc TGAGGCGGATAATCTCTCCAAATCACGTCGGCCTGACACTAGGGTTTATCTACCTTAC-- 523

**** ****** **** ***** * * * *********** *** ** *

R_ratt ACTATTTTATT 522

R_norv ACTATTTTATT 512

S_muel ACTATTTTATT 522

L_saba ACTATTCTATT 589

N_rapi ACTATTTTATT 520

A_flav ACCATTTTATT 520

A_sylv ACCATTTTATT 522

M_couc --CTTTGTATT 532

** ****

R_ratt - Rattus rattus, R_norv - Rattus norvegicus, S_muel - Sundamys muelleri, L_saba - Leopoldamys sabanus, N_rapi - Niviventer rapit, A_flav - Apodemus flavicollis, A_sylv - Apodemus sylvaticus, and M_couc - Mastomys coucha

Red nucleotides denote the site of target site duplication before insertion of the endogenous retrovirus-derived gene, 4632419I22Rik.
